# Vast Self-Renewal Potential of Human AGM Region HSCs Dramatically Declines in the Umbilical Cord Blood

**DOI:** 10.1016/j.stemcr.2020.08.008

**Published:** 2020-09-17

**Authors:** Andrejs Ivanovs, Stanislav Rybtsov, Richard A. Anderson, Alexander Medvinsky

**Affiliations:** 1 Ontogeny of Haematopoietic Stem Cells Group, Institute for Stem Cell Research, MRC Centre for Regenerative Medicine, University of Edinburgh, Edinburgh EH16 4UU, Scotland, UK; 2 Department of Morphology, Institute of Anatomy and Anthropology, Riga Stradiņš University, Riga LV-1010, Latvia; 3 School of Medicine, Dentistry and Nursing, College of Medical, Veterinary and Life Sciences, University of Glasgow, Glasgow G12 8QQ, Scotland, UK; 4 MRC Centre for Reproductive Health, Queen's Medical Research Institute, University of Edinburgh, Edinburgh EH16 4TJ, Scotland, UK

**Keywords:** AGM region, HSC, umbilical cord blood, human

## Abstract

Human hematopoietic stem cells (HSCs) emerge in the aorta-gonad-mesonephros (AGM) region during Carnegie stages (CS) 14–17. Although we previously reported that these HSCs can generate no less than 300 daughter HSCs, their actual number has never been established. Here, we show that a single human AGM region HSC can generate 600–1,600 functional daughter HSCs. The presence of HSCs in the CS 17 liver in one case gave us a unique opportunity to describe a reduction of HSC self-renewal potential after liver colonization. From a clinical perspective, the efficacy of long-term hematopoietic regeneration depends on HSC self-renewal capacity. We quantitatively show that this capacity dramatically declines in the umbilical cord blood compared with HSCs in the AGM region. A full appreciation of the vast regenerative potential of the first human embryo-derived HSCs sets a new bar for generation of clinically useful HSCs from pluripotent stem cells.

## Highlights

• A single human AGM region HSC can generate 600–1,600 fully functional daughter HSCs
• HSC self-renewal potential may decline by the time of liver colonization
• Transplanted umbilical cord blood HSCs self-renew without significant amplification
• This study sets a new bar for the self-renewal potential of human HSCs

## Teaser

In this article, A. Medvinsky and colleagues show that the first human hematopoietic stem cells (HSCs) emerging in the embryonic aorta-gonad-mesonephros region can generate up to 1,600 functional daughter HSCs upon transplantation into immunocompromized NSG mice. The vast self-renewal/regeneration potential of the first HSCs dramatically drops by the end of fetal development in umbilical cord blood HSCs.

## Introduction

Previous analyses using transplantations into adult NOD.Cg-*Prkdc*^*scid*^ *Il2rg*^*tm1Wjl*^/Sz (NSG) mice showed that the first human hematopoietic stem cells (HSCs) emerge in the aorta-gonad-mesonephros (AGM) region during Carnegie stages (CS) 14–17 (developmental days 32–41) and possess a substantial self-renewal potential (Ivanovs et al., 2011, 2014). However, the actual number of daughter HSCs could not be determined, as the dilution factor used has never reached the limit required to precisely quantify HSCs. Here, we have used a broader range of limiting dilutions to determine the accurate number of daughter HSCs generated by a single human embryo-derived HSC. We have also assessed the self-renewal potential of umbilical cord blood (UCB) HSCs, which are broadly used in clinical settings to treat congenital and acquired hematological disorders. This important quality of UCB HSCs has never been quantitatively assessed before.

## Results and Discussion

**Figure 1:**
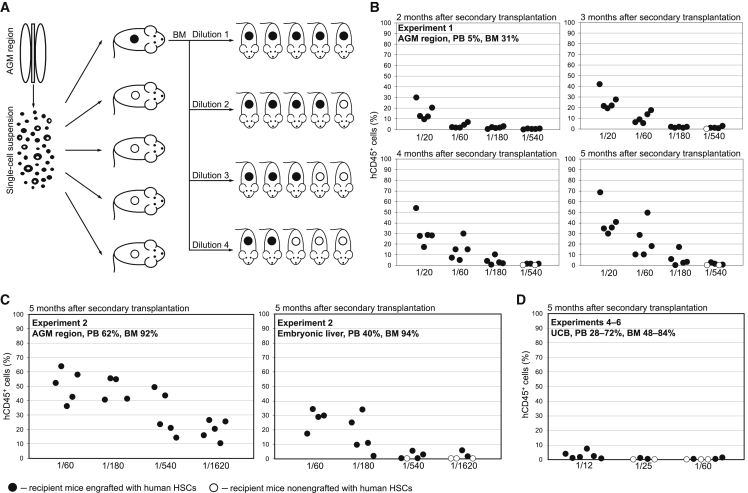
Self-renewal Capacity of Human AGM Region, Embryonic Liver, and UCB HSCs (A) A single-cell suspension prepared from an individual human AGM region was transplanted in equal portions into five primary NSG recipient mice. BM cells from the engrafted primary recipient were retranslated into four cohorts of secondary recipients at dilutions ranging from 1/20 to 1/1,620 of total primary BM per recipient. (B) Secondary repopulation dynamics over 5 months by BM cells from a primary NSG recipient reconstituted by a single AGM region HSC (experiment 1). (C) Secondary repopulation at 5 months by BM cells from primary NSG recipients reconstituted by a single AGM region HSC (left chart) or by liver HSCs (right chart; experiment 2). (D) Secondary repopulation at 5 months by BM cells from primary NSG recipients reconstituted by ≈10–20 UCB HSCs (50,000 CD34^+^ cells) each (cumulative of three independent transplantations). Human CD45^+^ cell chimerism was assessed in the PB of secondary recipients 2, 3, 4, and 5 months post transplantation. Circles represent individual NSG recipient mice. Tissue source and engraftment levels in primary NSG recipients (PB and BM) are indicated in the top left corner of each chart. Dilutions of primary BM cells transplanted per secondary recipient are indicated at the bottom of each chart. See also Tables S1 and S2 and Figure S1.

We performed three independent limiting-dilution transplantation experiments with human AGM region cells (Figure 1A). In each experiment, a single-cell suspension prepared from an individual AGM region was split into five equal parts and transplanted into five primary NSG recipient mice. Each time, human repopulation was found only in one out of five recipients, confirming that each AGM region contained only one definitive HSC (Ivanovs et al., 2011). To assess daughter HSC numbers generated by a single AGM region HSC, 5–9 months after the primary transplantation the bone marrow (BM) of each engrafted primary recipient was retransplanted in limiting dilutions into a cohort of 20 secondary recipients split into four groups. Mice in each group received cell doses ranging from 1/20 to 1/1,620 of total primary BM per recipient. The secondary recipients were monitored for 5 months and considered to be engrafted if at least 0.1% of peripheral blood (PB) CD45^+^ cells were of human origin belonging to both myeloid and lymphoid lineages.

In experiment 1, the AGM region (CS 16, developmental day 37) was transplanted. The engrafted primary recipient showed human multilineage hematopoietic chimerism (5% and 31% in PB and BM, respectively) (Table S1). Five months after the primary transplantation, serially diluted BM cells from the repopulated primary recipient were retransplanted into 20 secondary recipients. All secondary recipients that received 1/20, 1/60, or 1/180 of total primary BM showed human long-term multilineage PB engraftment (31% ± 14.0%, 14% ± 10.0%, and 3.6% ± 3.80%, respectively). Dilutions of 1/540 showed repopulation in only three of five secondary recipients, with markedly lower PB chimerism (1.1% ± 0.06%) (Figure 1B and Table S2). Using extreme limiting dilution analysis (ELDA) (Hu and Smyth, 2009), we calculated that a single human AGM region HSC produced ≈605 (95% CI: 254–1,440) daughter HSCs (Figure S1A).

**Figure 2:**
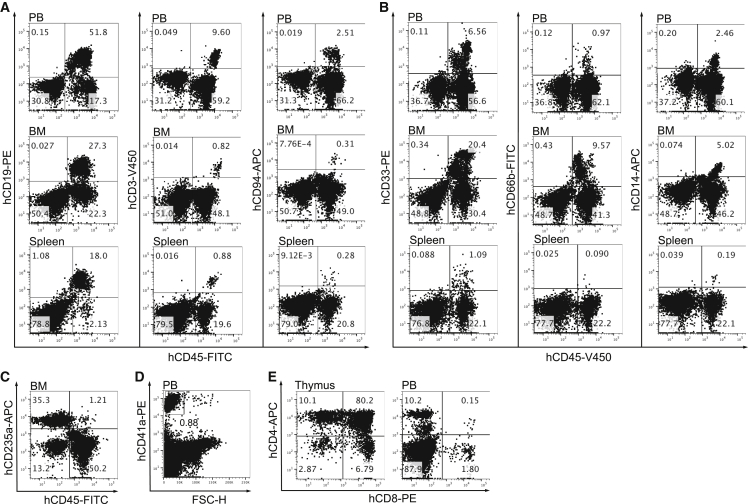
Human Long-Term Multilineage Hematopoietic Repopulation of a Primary NSG Recipient Mouse Engrafted with a Single AGM Region HSC (A) Human lymphoid (B, T, and NK cell) engraftment in the mouse PB, BM, and spleen. (B) Human myeloid (total, granulocyte, and monocyte) engraftment in the mouse PB, BM, and spleen. (C) Human erythroid engraftment in the mouse BM. (D) Human platelet engraftment in the mouse PB. (E) Human T cell populations in the mouse thymus and PB. These representative flow-cytometry plots correspond to experiment 2. Please note that on the plots showing BM repopulation, murine erythroid cells were not excluded, which explains the discrepancy with the percentage of human CD45^+^ cells reported in the text (92%). See also Table S1.

In experiment 2, the AGM region (CS 17, developmental day 41) was transplanted. The engrafted recipient showed high-level human multilineage chimerism (62% and 92% in PB and BM, respectively) (Table S1 and Figure 2). Six months after the transplantation, the BM from the primary repopulated recipient was retransplanted into secondary recipients. Five months later, all secondary recipients that received 1/60, 1/180, 1/540, and 1/1,620 dilutions showed human long-term multilineage PB chimerism (54% ± 16.1%, 44% ± 6.1%, 29% ± 12.2%, and 16% ± 6.8%, respectively) (Figure 1C, left chart, and Table S2). Saturated repopulation revealed that in this case the single AGM region HSC produced in excess of 1,620 daughter HSCs.

In experiment 3, the AGM region (CS 15, developmental day 33) transplantation resulted in high-level human multilineage hematopoietic engraftment of one recipient (PB and BM human chimerism 50% and 96%, respectively) (Table S1). Nine months later, the BM of this recipient was retransplanted. After 5 months, all secondary recipients transplanted with 1/60 and 1/180 primary BM dilutions showed human long-term multilineage PB chimerism (6.7% ± 7.51% and 5.0% ± 7.92%, respectively). Four of five secondary recipients transplanted with 1/540 dilutions showed 0.2% ± 0.07% PB chimerism. Dilutions of 1/1,620 gave no engraftment (only two of five secondary recipients survived) (Table S2). In this case, a single AGM region HSC produced ≈730 (95% CI: 313–1,699) daughter HSCs (Figure S1C).

Unexpectedly, the transplantation of CS 17 embryonic liver cells (from the embryo used in experiment 2) into two NSG recipients gave long-term multilineage hematopoietic repopulation in both mice. Previously, we observed a complete lack of HSCs in the liver up until at least CS 17 in 26 human embryos analyzed (Ivanovs et al., 2011). In this experiment, one of the two recipients showed PB and BM chimerism (40% and 94%, respectively) 6 months after transplantation and was used for a secondary transplantation experiment (Table S1). The other recipient initially showed multilineage engraftment, but later developed graft-versus-host disease and was excluded from further analysis. All secondary recipients that received 1/60 and 1/180 dilutions showed long-term multilineage PB chimerism (11% ± 3.7% and 3.8% ± 2.42%, respectively). Four of five secondary recipients transplanted with 1/540 dilutions showed PB chimerism (0.5% ± 0.39%). Only two of five secondary recipients transplanted with 1/1,620 dilutions showed low engraftment levels (0.3% ± 0.47%) (Figure 1C, right chart, and Table S2), suggesting that the liver-derived HSCs generated ≈880 (95% CI: 401–1,941) daughter HSCs during the 6 month period within the primary recipient (Figure S1B). This figure could be overestimated, as both primary recipients were repopulated and thus each could contain more than one liver HSC. Taken together, the self-renewal properties of liver HSCs were inferior to the AGM region HSCs of the same embryo.

We then assessed the self-renewal potential of human UCB HSCs. In two independent experiments, cohorts of five NSG recipients were transplanted with CD34^+^ cells isolated from fresh UCB using anti-human CD34 magnetic beads. Each primary recipient received 2,500 CD34^+^ cells, a dose containing only one or two HSCs (McDermott et al., 2010). In one experiment, all five recipients showed long-term engraftment 4 months after transplantation, and in the other experiment three of five recipients were engrafted (average PB chimerism was 0.6% ± 0.45%). At this point, we selected one primary recipient that showed the highest repopulation level from each experiment: mouse 1, with human PB and BM chimerism of 0.19% and 5.1%, respectively, and mouse 2, with PB and BM chimerism of 0.54% and 6.2%, respectively. The BM from these primary recipients was retransplanted independently into secondary recipients in dilutions of 1/20, 1/60, 1/180, and 1/540. Lack of engraftment in these two secondary transplantation experiments indicated that the dilution range of primary BM cells used in the experiments with AGM region cells appears to be too high to determine the self-renewal potential of UCB HSCs.

Therefore, we set up three additional independent experiments with higher UCB CD34^+^ cell doses and lower dilution ranges (experiments 4–6). In each experiment, 50,000 UCB CD34^+^ cells were transplanted per recipient, which is equivalent to 10–20 HSCs per mouse (McDermott et al., 2010). All primary recipients showed high-level PB engraftment (28%–72%) 5 months after transplantation (Table S1). From each experiment, the primary recipient with the highest human PB chimerism (72%, 33%, and 28%) was chosen. The BM of each primary recipient was then retransplanted in dilutions ranging from 1/12 to 1/60 (Figure 1D). All six secondary recipients transplanted with 1/12 dilutions showed an average PB chimerism of 2.7% ± 2.54%. Two of four secondary recipients transplanted with 1/25 dilutions showed an average PB chimerism of 0.3% ± 0.39%. Three of six secondary recipients transplanted with 1/60 of total primary BM per recipient showed an average PB chimerism of 0.3% ± 0.51% (Table S2). Statistically, this suggests that the transplantation of 10–20 UCB HSCs per recipient gave ≈33 (95% CI: 17–67) daughter HSCs per primary recipient 5 months later (Figure S1D). Thus, in striking contrast to AGM region HSCs, UCB HSCs possess much lower self-renewal capacity, enabling their maintenance without substantial expansion. Limited self-renewal capacity of UCB HSCs has been also reported by others, but using less efficient NOD/SCID recipients (Guenechea et al., 2001; Liu et al., 2010; McKenzie et al., 2006).

Overall, our study provides an accurate quantitative comparison between the self-renewal potential of the first emerging HSCs in the human embryo and those in the UCB at birth. The actual production of daughter HSCs by an individual AGM region HSC reaches 600–700, and in one case was more than 1,620. The differences observed between embryos likely arise from genetic variability. Possibly not by coincidence, the embryo whose AGM region HSCs showed the highest daughter HSC production also showed unusually early HSC colonization of the embryonic liver. By direct comparison of AGM region and liver HSCs with the same genetic background, we found that both the repopulation levels and the self-renewal capacity of liver HSCs were inferior compared with the AGM region HSCs (880 versus 1,620 daughter HSCs, respectively). Previously, except for one case of multilineage long-term engraftment by cultured CS 17 liver (Easterbrook et al., 2019), liver cell transplantations from 26 human embryos up to CS 17 gave no repopulation. A dramatic decline in the self-renewal capacity is clearly observed in UCB HSCs compared with AGM region HSCs. Whether this has already started in the liver requires further systematic analysis at later developmental stages. Notably, the repopulation potential of mouse HSCs declines between the fetal liver and the adult BM stages (Holyoake et al., 1999; Nicolini et al., 1999).

Adult BM and UCB HSCs are successfully used in clinical transplantations (Gluckman et al., 1989; Good et al., 1969). To enhance the safety and predictability of clinical HSC transplantations, there is a need to generate readily available human HSCs (Daley, 2007). HSC expansion was achieved *in vitro* (Fares et al., 2014; Gupta et al., 2020; Wilkinson et al., 2019), and long-term repopulating hematopoietic cells were produced from pluripotent stem cells and endothelial cells using gene manipulations (Ivanovs et al., 2017). Our study sets a new bar for the self-renewal potential of human HSCs that can be generated *in vitro*. Molecular mechanisms underlying the exceptional regenerative capacity of human AGM region HSCs require further elucidation.

## Experimental Procedures

### Human Embryonic Tissues and Umbilical Cord Blood

Human embryonic tissues were obtained immediately after elective termination of pregnancy using mifepristone and misoprostol. The study was approved by the Lothian Research Ethics Committee. Before tissue specimens were obtained, each patient gave informed consent for the use of embryonic tissues. The developmental stage of human embryos was determined according to the Carnegie staging system (O’Rahilly and Müller, 1987). Embryonic tissue dissection and single-cell suspension preparation were performed as previously described (Ivanovs et al., 2011). Fresh UCB was obtained from the Edinburgh Reproductive Tissue Biobank. Ficoll-Paque (STEMCELL Technologies) was used to separate mononuclear cells. CD34^+^ cells were isolated by employing the Miltenyi Biotec magnetic cell separation technique.

### Animals

NSG mice were bred within the University of Edinburgh (Edinburgh, UK) according to the provisions of the Animal (Scientifi Procedures) Act 1986 under the project licence granted by the Home Office (UK). Mice were kept under specific-pathogen-free conditions in individually ventilated cages. Up to 6 h before transplantation with human cells, 6–8 week old female NSG mice received a sublethal total body irradiation dose of 3.5 Gy at a rate of 0.75 Gy/min from a ^137^Cs source (GSR D1 γ-irradiator, Gamma-Service Medical). Animals were transplanted with cells intravenously via the tail vein.

### Primary Murine Recipient Bone Marrow

The BM was flushed out from the bones and a single-cell suspension was prepared by gentle pipetting. A cell fraction associated with endosteum was obtained from bones after treatment with 1 mg/mL collagenase/dispase and 0.12 mg/mL DNase I at 37°C for 40 min with gentle shaking and added to the flushed-out BM fraction. To calculate the proportion of total BM transplanted into secondary recipients, we used previously reported data on the distribution of BM cells in different mouse bones (Boggs, 1984).

### Flow-Cytometry Analysis

The following mouse anti-human monoclonal antibodies (all from BD) were used for flow-cytometry analysis: CD3-APC, PE and PerCP (clones SK7 and SP34-2), CD4-APC and APC-Cy7 (clone RPA-T4), CD8-PE and PE-Cy7 (clone RPA-T8), CD11b-PE-Cy7 (clone ICRF44), CD13-APC (clone WM15), CD14-APC and APC-Cy7 (clones M5E2 and MϕP9), CD19-PE (clone HIB19), CD33-PE (clone WM53), CD34-APC (clone 8G12), CD38-FITC and PE (clone HIT2), CD41a-FITC (clone HIP8), CD45-Biotin, FITC, and V450 (clone HI30), CD66b-FITC (clone G10F5), CD94-APC (clone HP-3D9), CD235a-APC (clone GA-R2), IgM-Biotin (clone G20-127), αβ TCR-FITC (clone WT31), and γδ TCR-PE (clone 11F2). The Human FcR Blocking Reagent (Miltenyi Biotec) and anti-mouse CD16/32 purified monoclonal antibody (clone 93) (eBioscience) were used to prevent unwanted binding of antibodies to Fc receptors. All antibodies and reagents listed above were used at final concentrations either recommended by the manufacturer or determined by titration in-house. 7-Amino-actinomycin (eBioscience) was used for dead cell exclusion. A FACSCalibur, LSRFortessa (both from BD), or CyAn ADP (Dako) instrument was used for flow-cytometry analysis. Flow-cytometry data were analyzed with FlowJo v.7.6.1 software (Tree Star).

### Statistical Analysis

Data are presented as a mean ± SD or a 95% CI. Validity tests for the single-hit Poisson model and the limiting dilution analysis were performed using ELDA software (Hu and Smyth, 2009) available at http://bioinf.wehi.edu.au/software/elda/ (Figure S1).

## Author Contributions

The experimental part of this study was performed at the University of Edinburgh (Edinburgh, UK). A.I. and S.R. performed the experiments, interpreted experimental data, and wrote the manuscript. R.A.A. provided human embryonic material and feedback on data analysis. A.M. directed the study, interpreted experimental data, and wrote the manuscript.

## Supplemental Information

Document S1. Figures S1

Table S1. Primary Recipient Engraftment Data, Related to Figures 1 and 2

Table S2. Secondary Recipient Engraftment Data, Related to Figure 1

Document S2. Article plus Supplemental Information
