# Supplementary material for: Vast Self-Renewal Potential of Human AGM Region HSCs Dramatically Declines in the Umbilical Cord Blood
Source: Stem Cell Reports. 2020 Sep 17;15(4):811–6. doi: 10.1016/j.stemcr.2020.08.008 (PMC7561509; doi:10.1016/j.stemcr.2020.08.008)
Supplement: Document S1. Figures S1 [file mmc1.pdf]

Stem Cell Reports, Volume *15*

Supplemental Information

Vast Self-Renewal Potential of Human AGM Region HSCs Dramatically Declines in the Umbilical Cord Blood

Andrejs Ivanovs, Stanislav Rybtsov, Richard A. Anderson, and Alexander Medvinsky

A

BM dilutions (1/20, 1/60, 1/180, 1/540)

Counter	Dose	Tested	Response
1	5125000	5	5
2	1708333	5	5
3	569444	5	5
4	189815	5	3

Number of BM cells containing one HSC

Lower	Estimate	Upper
404432	169692	71200

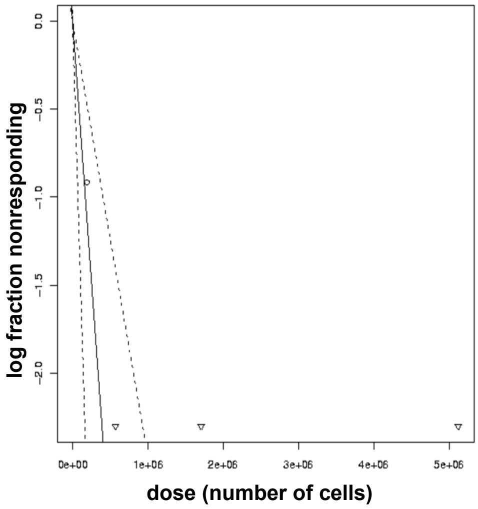

B

BM dilutions (1/60, 1/180, 1/540, 1/1,620)

Counter	Dose	Tested	Response
1	1541667	5	5
2	513889	5	5
3	171296	5	4
4	57098	5	2

Number of BM cells containing one HSC

Lower	Estimate	Upper
230372	104749	47629

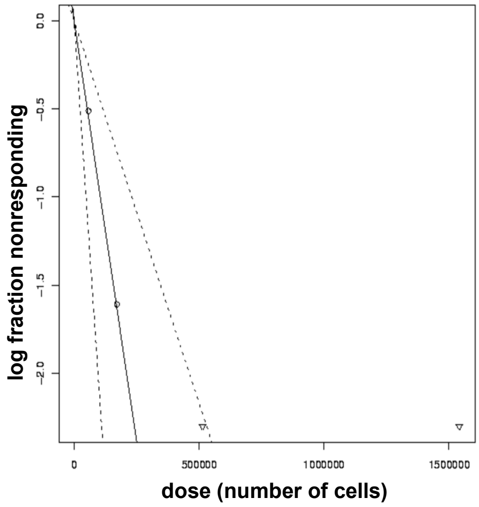

C

BM dilutions (1/60, 1/180, 1/540, 1/1,620)

Counter	Dose	Tested	Response
1	2666667	5	5
2	888889	5	5
3	296296	5	4
4	98765	2	0

Number of BM cells containing one HSC

Lower	Estimate	Upper
511826	219515	94147

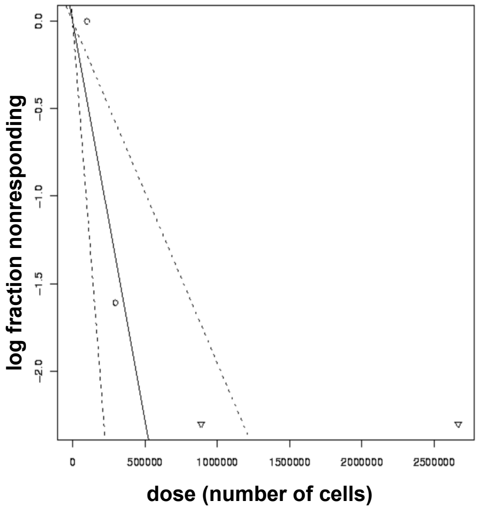

D

BM dilutions (1/12, 1/25, 1/60)

Counter	Dose	Tested	Response
1	7500000	6	6
2	3600000	4	2
3	1500000	6	3

Number of BM cells containing one HSC

Lower	Estimate	Upper
5397938	2712349	1362898

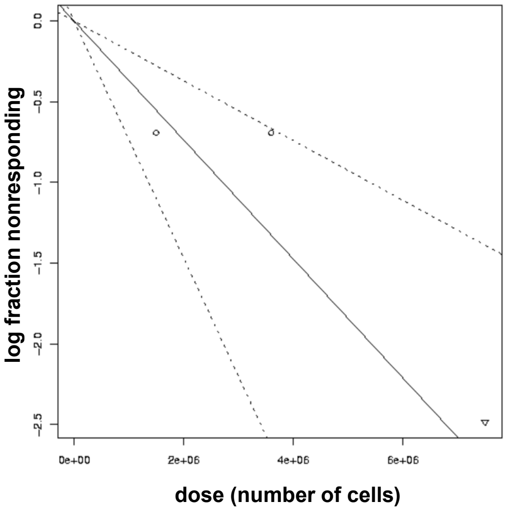

Figure S1. Calculation of Daughter HSC Numbers. Related to Figure 1.
(A) Experiment 1 (AGM region). Number of harvested BM mononuclear cells: 41,000,000. Estimated total number of BM mononuclear cells: 102,500,000.
(B) Experiment 2 (Liver). Number of harvested BM mononuclear cells: 37,000,000. Estimated total number of BM mononuclear cells: 92,500,000
(C) Experiment 3 (AGM region). Number of harvested BM mononuclear cells: 54,000,000. Estimated total number of BM mononuclear cells: 160,000,000.
(D) Experiments 4–6 (UCB). Number of harvested BM mononuclear cells: 36,000,000. Estimated total number of BM mononuclear cells: 90,000,000.
ELDA is a software application for limiting dilution analysis in extreme data situations when some data sets give zero negative or positive responses. The method is particularly suitable for stem cell analysis. The bone marrow from each primary recipient was harvested from 12 bones (two femora, tibiae, fibulae, humeri, radii, and ulnae) and pelvis. The total number of mononuclear cells in the recipient was calculated assuming that the proportion of BM harvested represents ≈40% of total BM (Boggs, D.R. (1984). The total marrow mass of the mouse: a simplified method of measurement. Am J Hematol *16*, 277-286). For each experiment, the first table is populated with cell numbers corresponding to different dilutions (dose), total number of transplanted mice for each dilution (tested), and number of engrafted mice (response). The second table shows the estimated number of BM mononuclear cells containing one HSC and the 95% CI (lower and upper). The total number of HSCs in primary recipients was calculated by dividing total BM mononuclear cell number by the cell number containing one HSC. In the graphs, the natural logarithm of the nonengrafted mice fraction was plotted against the cell dose transplanted per recipient (shown by open circles). Down-pointing triangles represent the data value with zero negative or positive responses (all recipients for the same dilution are either nonengrafted or engrafted). The solid diagonal line indicates the mean HSC number per cell dose transplanted and dotted lines show the 95% CI. HSC numbers transplanted per recipient mouse (λ) can be calculated from the formula $\lambda = -\ln(\text{fraction of nonengrafted mice})$. From here, $-\ln(0.37) \approx 1$, which means that the dose of transplanted cells yielding ≈37% of nonengrafted recipient mice contains ≈1 HSC. The cell dose containing one HSC can be determined on the graph by plotting an imaginary horizontal line from the y axis at –1 to intercept the solid diagonal line and then by drawing a vertical line from the point of interception of the horizontal line and the solid diagonal line until it cuts the x axis.

Supplemental Table Captions

Table S1. Primary Recipient Engraftment Data. Related to Figure 1 and Figure 2.

Table S2. Secondary Recipient Engraftment Data. Related to Figure 1.
